# Subjective health status, life performance and complications in chronic hypoparathyroidism – a German multicenter survey

**DOI:** 10.3389/fendo.2026.1723640

**Published:** 2026-03-31

**Authors:** Carmina T. Fuss, Sarah Engbers, Nicole Reisch, Anke Hannemann, Henry Völzke, Hans J. Grabe, Matthias Nauck, Michael Droste, Holger S. Willenberg, Nada Rayes, Martin Fassnacht, Marcus Quinkler, Stefanie Hahner

**Affiliations:** 1 Department of Internal Medicine, Division of Endocrinology and Diabetes, University Hospital, University of Wuerzburg, Wuerzburg, Germany; 2 Medizinische Klinik und Poliklinik IV, Klinikum der Universität München, Munich, Germany; 3 Institute of Clinical Chemistry and Laboratory Medicine, University Medicine Greifswald, Greifswald, Germany; 4 DZHK (German Center for Cardiovascular Research), partner siteGreifswald, Greifswald, Germany; 5 Institute for Community Medicine, University Medicine Greifswald, Greifswald, Germany; 6 Department of Psychiatry and Psychotherapy, University Medicine Greifswald, Greifswald, Germany; 7 German Centre for Neurodegenerative Diseases (DZNE), Partner SiteRostock/Greifswald, Greifswald, Germany; 8 Practice for Endocrinology and Diabetes, Oldenburg, Germany; 9 Division of Endocrinology and Metabolism, Rostock University Medical Center, Rostock, Germany; 10 Department of General, Visceral, and Transplant Surgery, University Hospital Leipzig, Leipzig, Germany; 11 Endokrinologie in Charlottenburg, Endokrinologie Praxis am Stuttgarter Platz, Berlin, Germany

**Keywords:** burden of illness, comorbidities, hypoparathyroidism, quality of life, Short Form-36

## Abstract

**Background:**

There is mounting evidence that conventional replacement strategies with calcium and vitamin D are insufficient to fully prevent complications of chronic hypoparathyroidism (HypoPT) in all patients.

**Methods:**

To investigate the disease burden of chronic HypoPT, we performed a survey in 205 HypoPT patients (159 females; median age 55 years; median disease duration 16 years). Patients received a disease-specific questionnaire asking for subjective health status, comorbidities, disease-related emergencies and interference of HypoPT with daily and work life. Patients were further assessed by the SF-36 questionnaire. Data was compared to sex- and age-matched subjects from the Study of Health in Pomerania SHIP-START, the German Health Interview and Examination Survey for Adults (DEGS1) and to 214 patients with adrenal insufficiency.

**Results:**

Clinical symptoms associated with HypoPT during the past 12 months were reported by 92% of patients, requiring medical intervention in 32%. Since primary diagnosis of HypoPT, 36% of patients had presented at least once at an emergency department due to severe hypocalcemia (14.7 events per 100 patient years). Trigger factors for hypocalcemic symptoms were reported by 76% of patients (e.g. physical activity, infections, hot weather). In comparison to population-based controls, patients with HypoPT showed a higher prevalence of renal insufficiency (11% vs. 2%, p<0.001) and more frequently received antihypertensive (44% vs. 34%; p=0.003) and antiepileptic drugs (5% vs. 2%, p=0.01). Lifetime prevalence of both depression (22% vs. 15%, p=0.003) and anxiety (21% vs. 6%, p<0.001) were increased in HypoPT. SF-36 values indicated significantly reduced subjective health status in HypoPT patients compared to controls as well as patients with adrenal insufficiency.

**Conclusion:**

Despite established treatment, chronic HypoPT is associated with a variety of symptoms with a significant impact on daily life and work life.

**Trial registration:**

https://ClinicalTrials.gov/NCT03437174.

## Introduction

Chronic hypoparathyroidism (HypoPT) is a rare disorder caused by insufficient secretion of parathyroid hormone (PTH) or a reduced receptor effect of the hormone (1, 2). HypoPT is most commonly iatrogenic, resulting from thyroid or other neck surgery (3). Unlike other hormonal deficiencies, HypoPT is usually not treated by replacement of the missing hormone. The widely accepted long-term conventional treatment with calcium and vitamin D does, however, not completely restore calcium and phosphorus homeostasis in a physiological manner (1, 4). Even though treatment with calcium and vitamin D can certainly prevent severe hypocalcemia and reduces tetanic symptoms, there is growing evidence that subjective health status (SHS) is not fully restored under this replacement regimen (5). In addition, patients experience a broad spectrum of immediate symptoms and long-term complications directly related to impaired calcium homeostasis (1, 6, 7). In a chart review of 120 patients with permanent HypoPT, renal calcifications were detected in 31% of patients by imaging and stage 3–5 chronic kidney disease was 2- to 17-fold more prevalent compared to age-matched norms from the National Health and Nutrition Examination Survey (8, 9). Finally, the risk for renal insufficiency, cardiovascular disease, neuropsychiatric complications, infections, seizures, cataract and fractures has been reported to be increased in HypoPT patients (10–14).

Few studies have assessed the SHS in patients with HypoPT so far. A German cross-sectional study investigating 25 female patients with postsurgical HypoPT on stable treatment with calcium and vitamin D showed a significantly reduced SHS and impaired mood in comparison to 25 controls with a history of thyroid surgery but intact parathyroid function (5). In a more recent web-based survey including 374 U.S. residents with HypoPT, many patients reported a significant interference of HypoPT with their daily life and work life due to HypoPT and a high percentage indicated feelings of anxiety and depression during the past 12 months (6). A Danish Study comparing 22 patients with postsurgical HypoPT and hypothyroidism to 22 patients with only postsurgical hypothyroidism provided evidence that combined postsurgical HypoPT and hypothyroidism is associated with a more severe impairment of quality of life measured by Short Form-36 (SF-36) compared to hypothyroidism with intact parathyroid function (15). Moreover, patients with postsurgical HypoPT and hypothyroidism scored clearly worse than healthy controls, indicating relevantly reduced quality of life. Recently, an analysis of 283 patients with HypoPT from Norway also revealed a significant reduction of SHS in the self-assessment questionnaires SF-36 and HADS compared to unmatched normative data from the Norwegian population (16). A 13-country patient and caregiver survey revealed substantial disease burden in 398 patients with inadequately controlled hypoparathyroidism (17). Several case reports and studies furthermore indicate an increased risk for psychiatric complications in patients affected by HypoPT in terms of anxiety and depression (18–20).

In the present study, we aimed to investigate the burden of disease in a large cohort of patients with HypoPT in comparison to well-characterized age- and sex-matched controls from the background population. We particularly addressed SHS and the resulting impact of HypoPT on daily life and work life, the prevalence of comorbidities, and emergency events due to HypoPT.

## Patients and methods

### Patients

Patients with documented chronic HypoPT and pseudo-HypoPT were included based on the following criteria: Established replacement therapy, defined as a regimen consisting of at least one of the following medications: vitamin D or its metabolites and analogs, calcium supplements or subcutaneous rhPTH. Patients with pseudo-HypoPT were included because, despite differences in underlying pathophysiology, they share chronic hypocalcemia, long-term treatment requirements, and a comparable disease burden with patients affected by classical hypoparathyroidism. In addition, disease duration of at least 12 months and age above 18 years was required. Patients from five participating institutions across Germany were included: 4 tertiary care centers (University Hospitals Munich, Berlin, Rostock and Wuerzburg) as well as 2 endocrine practices (Berlin and Oldenburg). In addition, members of the German Self-Help Network of patients with chronic HypoPT received written information on the survey and were invited to participate.

All patients with documented diagnosis of HypoPT treated at the University Hospitals of Wuerzburg, Munich and Rostock were contacted by mail. Patients at the University Hospital in Berlin were contacted by phone and invited to participate in the study. In the two private practices, patients received the questionnaires at their outpatient visit. Members of the Self-Help Network were invited via the website to send their contact data to study organizers if interested in participation.

The study was approved by the ethical committee of the University of Wuerzburg (AZ134/14). All participants gave written informed consent (ClinicalTrials.gov identifier: NCT03437174).

### Questionnaires

All study participants received a disease-specific questionnaire and the validated self-assessment SF-36 (version 1.0) (21). The disease-specific questionnaire was developed at the University Hospital in Wuerzburg (**Supplementary 1**). The 16 questions focused on duration, cause and symptoms of HypoPT, symptoms of hypocalcemia, factors enhancing those symptoms, self-perceived health status, influence of HypoPT on daily life and work life, comorbidities, medication, intercurrent illness, episodes of severe hypocalcemia, need of intravenous calcium supplementation and hospital admission. Besides multiple-choice items the questionnaire contained free-text fields for additional comments. 11 questions of the disease specific questionnaire were kept identical to those used in the population-based surveys used for comparison.

SHS was evaluated by the validated self-assessment questionnaire SF-36 (21). The SF-36 consists of 36 items representing eight domains of physical and mental health: perception of physical functioning (PF), role limitations due to physical problems (RP), bodily pain (BP), general health perception (GH), vitality (VT), social functioning (SF), role limitations due to emotional problems (RE) and general mental health (MH). The domain scores range from 0-100 with high scores indicating a more favorable health related quality of life.

### Controls

Data were compared to sex- and age-matched control groups from two population-based cohorts: the German Health Interview and Examination Survey for Adults (DEGS1) (22) and the Study of Health in Pomerania (SHIP)-START. Hypoparathyroidism was not assessed in the control groups, based on the assumption of a very low disease prevalence in the general population. The DEGS1 was conducted by the Robert Koch Institute (RKI), Berlin from 2008–2011 and included 8152 subjects between 18–79 years from the general German population. The public use file comprises 7987 subjects who participated in an Interview Survey. Of these participants, 7115 had also participated in an Examination Survey. The comparison between the HypoPT patients and the DEGS1 participants followed an individual matching for sex and age (by decade). Based on the highest possible number of matchings, a 1:20 matching was realized for the Interview Survey and a 1:17 matching for the combined Examination and Interview Survey by randomly selecting controls in the same sex- and age-strata. Data regarding self-reported diagnosis of anxiety or depression, mental health status, perception of general health and impairment in daily life were compared between the groups. Regarding work life impairment, 157 HypoPT participants younger than 65 years were matched at a ratio of 1:20 to controls aged below 65 years (n=5938 of all DEGS1 participants). Moreover, 162 HypoPT patients after thyroid surgery were compared to 162 sex- and age-matched DEGS1 participants with self-reported history of thyroid surgery (out of 366 eligible patients after thyroid surgery in the DEGS1 group).

SHIP-START is a population-based cohort study located in northeast Germany (23, 24). Baseline examinations (SHIP-START-0) were performed from 1997–2001 in 4308 adult men and women, followed by 5-year (SHIP-START-1) and 11-year follow-up examinations (SHIP-START-2). In all SHIP waves, standardized medical examinations including blood pressure measurement, blood sampling and an extensive computer-aided personal interview were offered to the participants. Data on socio-demographic characteristics, lifestyle and medical histories was collected. SHIP-START-2 was conducted between 2008–2012 with 2333 participants aged 30 years or older. The Composite International Diagnostic-Screener (CID-S) was used to obtain information on self-reported experience of loss of interest and energy, sadness, tension and anxiety attack (25). These data were compared with the HypoPT cohort. For this, sex- and age-matched controls were selected from SHIP-START-2 to the 196 HypoPT subjects older than 30 years. Each HypoPT patient was matched to 6 randomly selected controls from the same sex- and age-strata in SHIP-START-2.

Reference data for SF-36 scores were obtained from the German National Health Survey (BGS98) (26) and from patients with chronic primary or secondary adrenal insufficiency (AI) (27). The German National Health Survey was performed from 1997–1999 by the RKI and includes data of a representative random sample of 7124 subjects from the general German population.

### Statistical analysis

SPSS version 23.0 (IBM), was used for statistical analysis. Characteristics of the HypoPT patients are presented as median with range (continuous data) or number with proportion (categorical data). Group differences between HypoPT patients and the matched controls from DEGS1 or SHIP-START-2 controls were performed using either McNemar tests (dichotomous variables) or Friedman’s Chi-Square Test (categorical variables). SF-36 scores of HypoPT patients were compared to those obtained in the BGS98 and in patients with AI. As sex- and age-structure differ between the cohorts, the SF-36 values of each cohort were transformed into age- and sex-adjusted z-scores. Group differences in z-scores were then assessed using Mann-Whitney U tests. Finally, Pearson’s correlation coefficients were calculated to investigate the relationship between disease duration of HypoPT and different domains of SF-36. No formal adjustment for multiple comparisons was applied, as the analyses were exploratory in nature. A p-value <0.05 was considered statistically significant.

## Results

### Study cohort

412 patients were contacted for participation with a response rate of 52% (n=215). Ten patients did not fulfill all inclusion criteria; therefore, 205 patients were included for statistical evaluation (Berlin University Hospital (Charite) n=8, Berlin endocrine practice n=30, Munich University Hospital n=34, Oldenburg endocrine practice n=21, Rostock University Medical Center n=10, self-help network n=32, Wuerzburg University Hospital n=70).

General characteristics of the HypoPT cohort are given in **Table 1**. The age distribution was equal in patients with postsurgical (n=176), non-surgical HypoPT (n=19) and pseudo-HypoPT (n=10), whereas the sex distribution and the disease duration differed significantly between etiologies. Median disease duration in patients with pseudo-HypoPT was 51 years (range: 25–65 years) and thereby clearly longer than in patients with non-surgical and postsurgical HypoPT with 16 years (range: 3–57 years) and 10 years (range: 1–60 years), respectively (**Table 1**). A total of 2,930.28 patient-years were recorded in the study.

**Table 1:** General characteristics of patients with hypoparathyroidism (HypoPT).

Sex n (%)^+^	46 male (22) 159 female (78)
Age (years) Median (range)	55 (24-84)
Disease duration (years)° Median (range)	16 (1-65)
Cause of HypoPT	n (%)
Postsurgical HypoPT due to surgery for:	176 (86)
Goiter	98 (48)
Thyroid cancer	48 (23)
Grave´s disease	17 (8)
Primary Hyperparathyroidism	11 (5)
Riedel thyroiditis	1 (0.5)
Thyroid cysts	1 (0.5)
Non-surgical HypoPT	19 (9)
Autoimmune HypoPT	4 (2)
Congenital HypoPT	4 (2)
Idiopathic HypoPT	11 (5)
Pseudohypoparathyroidism	10 (5)
Member self-helping group n (%)	32 (16)
Medication of HypoPT	n (%)
Oral calcium supplements	159 (78)
Active vitamin D metabolites	187 (91)
Calcitriol	113 (55)
Alphacalcidiol	44 (21)
Dihydrotachysterol	30 (15)
Vitamin D2/D3	62 (30)
Magnesium	79 (39)
Subcutaneous PTH 1-34	3 (1%)
Experienced symptoms of HypoPT in last 12 months	% of patients *
Paraesthesia	71
Carpopedal spasm	60
Muscle pain	44
Increased irritability	35
Bone pain	34
Anxiety	31
Muscle weakness	28
Enterospasm	19
Seizure	7
Observed trigger factors for symptoms of hypocalcemia	% of patients
Physical activity	42
Perspiration	30
Gastrointestinal complaints	29
Hot weather	25
Infectious disease	23
Stressful events	14

^+^proportion of female patients: postoperative hypoPT 81%, non-postoperative hypoPT 63.2%, pseudohypoPT 40% (p<0.05).

°median disease duration (years): postoperative hypoPT 9.9, non-postoperative hypoPT 15.5, pseudohypoPT 51.1 (p<0.05).

*percentage of patients experiencing respective symptom within the last 12 months.

### Comorbidities, hypocalcemic events and medical care

92% of HypoPT participants reported to have suffered from at least one clinical symptom potentially associated with HypoPT during the past 12 months, most commonly being paraesthesia (71%), carpopedal spasms (60%), muscle pain (44%) and increased irritability (35%) (**Table 1**).

76% of patients reported that symptoms of hypocalcemia became more pronounced in specific situations. The most common trigger factor was physical activity (42%), followed by perspiration (30%), gastrointestinal events (29%), hot weather (25%) and infections (23%) (**Table 1**).

**Figure 1:**
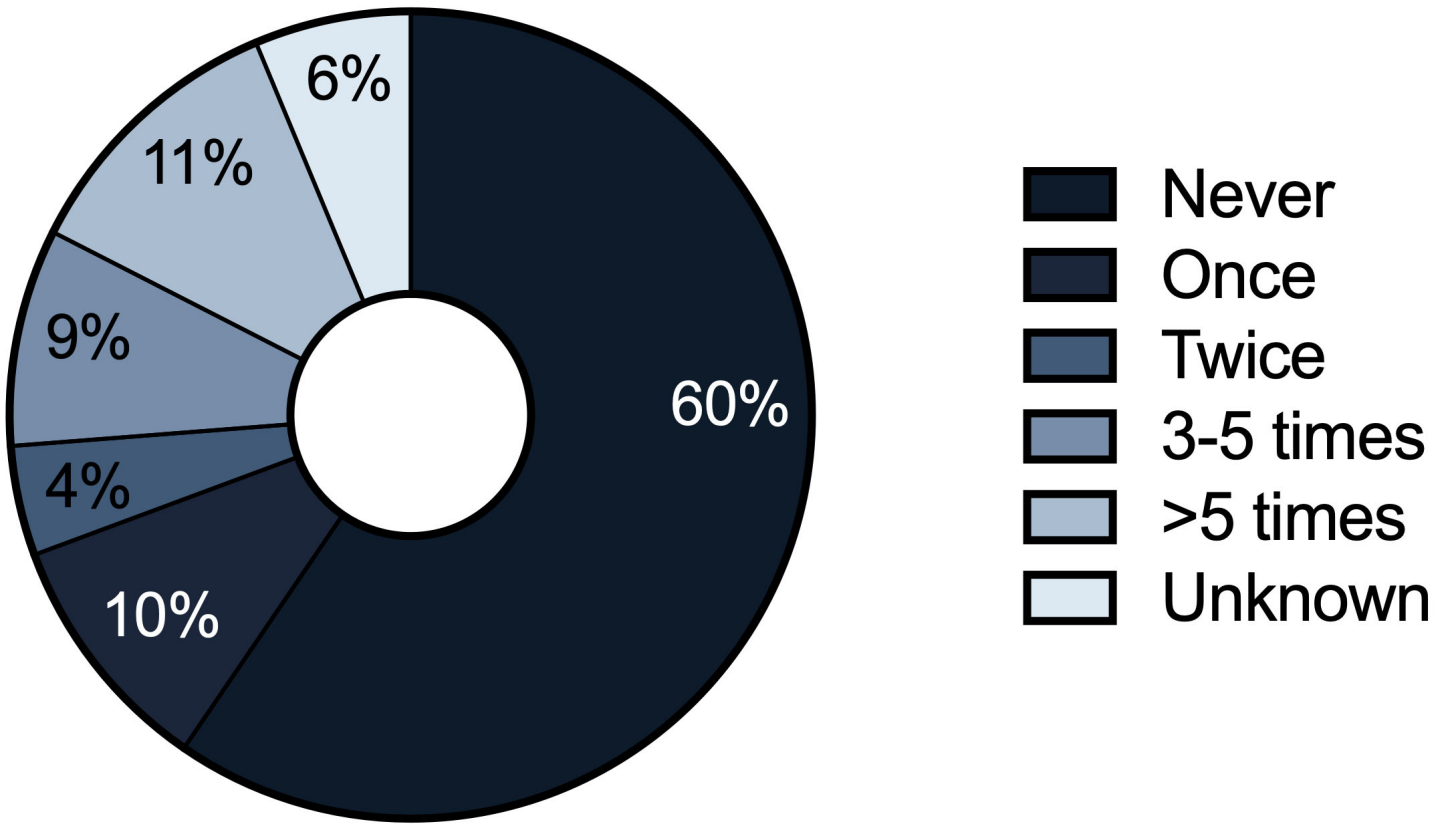
Frequency of intravenous calcium administration since primary diagnosis of hypoparathyroidism among the HypoPT patients (n=205).

Since diagnosis of HypoPT, 36% of patients had presented at least once at an emergency department or were admitted to the hospital because of severe symptoms caused by HypoPT. Furthermore, 70 patients (34%) received intravenous calcium at least once due to severe symptoms of hypocalcemia and 11% were treated with intravenous calcium more than six times since primary diagnosis (**Figure 1**). Overall frequency of hypocalcemic emergencies requiring intravenous calcium supplementation was 14.7 events per 100 patient years. General hospitalization rate of HypoPT patients in the past 12 months was, however, not significantly different from controls of the DEGS1 (19% vs. 15%, p=0.145).

Lifetime prevalence of renal insufficiency reported by patients in the interview was significantly higher in HypoPT compared to the age-matched DEGS1 population (11% vs. 2%, p<0.001). Significantly more HypoPT patients were treated with antihypertensive agents (44% vs. 34%, p=0.003) or antiepileptic agents (5% vs. 2%, p=0.01).

**Figure 2:**
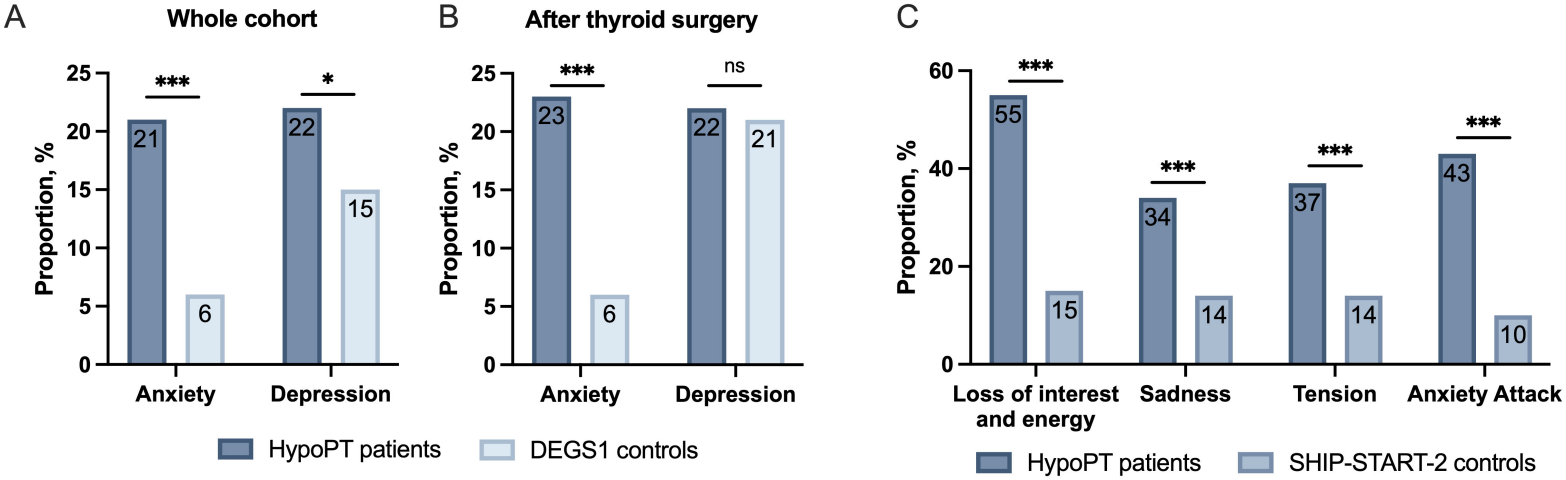
**(A)** Frequency of anxiety and depression in patients with hypoparathyroidism (HypoPT, n=202) compared to controls from DEGS1 (n=4040). **(B)** Frequency of anxiety and depression in patients with hypoparathyroidism due to thyroid surgery (n=162) compared to controls from DEGS1 after thyroid surgery (n=162). **(C)** Comparison of mental health status regarding anxiety, depression, and tension during the last 12 months between patients with hypoparathyroidism (HypoPT, n=196) and controls from SHIP-START-2 (n=1176). *p<0.05, ***p<0.001, ns, not significant.

Regarding psychiatric comorbidities, lifetime prevalence of both depression (22% vs. 15%, p=0.003) and anxiety (21% vs. 6%, p<0.001) were higher in HypoPT (**Figure 2A**). Patients affected by HypoPT after thyroid surgery also showed a significantly higher rate of anxiety than controls after thyroid surgery (23% vs. 6%, p<0.001) (**Figure 2B**). Furthermore, significantly more patients with HypoPT reported feelings of sadness, tension, anxiety attacks or loss of interest and energy during the last 12 months compared to the general population (SHIP-START-2) (**Figure 2C**).

Regarding concomitant medication, intake of antihypertensive medication and medication affecting the cardiovascular system was significantly increased in patients with HypoPT compared to the general population. Significantly more patients with HypoPT were treated with beta blockers, diuretics and medication affecting the renin-angiotensin-aldosterone-system (**Table 2**).

**Table 2:** Concomitant medication affecting the cardiovascular system.

Medication	HypoPT n=202	DEGS1 controls n=3434	P-value
Betablockers, n (%)	50 (25)	652 (19)	0.046
Calcium channel blockers, n (%)	14 (7)	232 (7)	0.933
Diuretics, n (%)	30 (15)	194 (6)	<0.001
Medication affecting the RAAS, n (%)	63 (31)	796 (23)	0.01
Lipid agents, n (%)	21 (10)	401 (12)	0.6

RAAS, Renin-angiotensin-aldosterone-system.

### Subjective health status

SF-36 values were significantly decreased in seven out of eight domains in patients affected by HypoPT compared to the general German population (all p<0.001, except RE with p=0.017). Only bodily pain showed no significant differences (p=0.167). A higher number of HypoPT associated experienced symptoms was related to a stronger impairment of SHS in all domains (all p<0.001, except RE p=0.004). However, there was no correlation between disease duration of HypoPT and any domain from the SF-36 (physical functioning: r=0.048, p=0.503, role limitations due to physical problems: r=0.056, p=0.435, bodily pain: r=0.02, p=0.695, general health perception: r=0.05, p=0.453, vitality: r=0.063, p=0.37, social functioning: r=0.048, p=0.491, role limitations due to emotional problems: r=0.08, p=0.267, general mental health: r=-0.013, p=0.859). Members of the Self-Help Network compared to patients without membership scored significantly worse in the domains of general health perception (p=0.04) and social functioning (p=0.004). Additionally, there were no significant differences regarding the SF-36 between participants of University Hospitals and private practices, as well as between different etiologies of HypoPT (postsurgical, non-surgical and pseudo-HypoPT).

**Figure 3:**
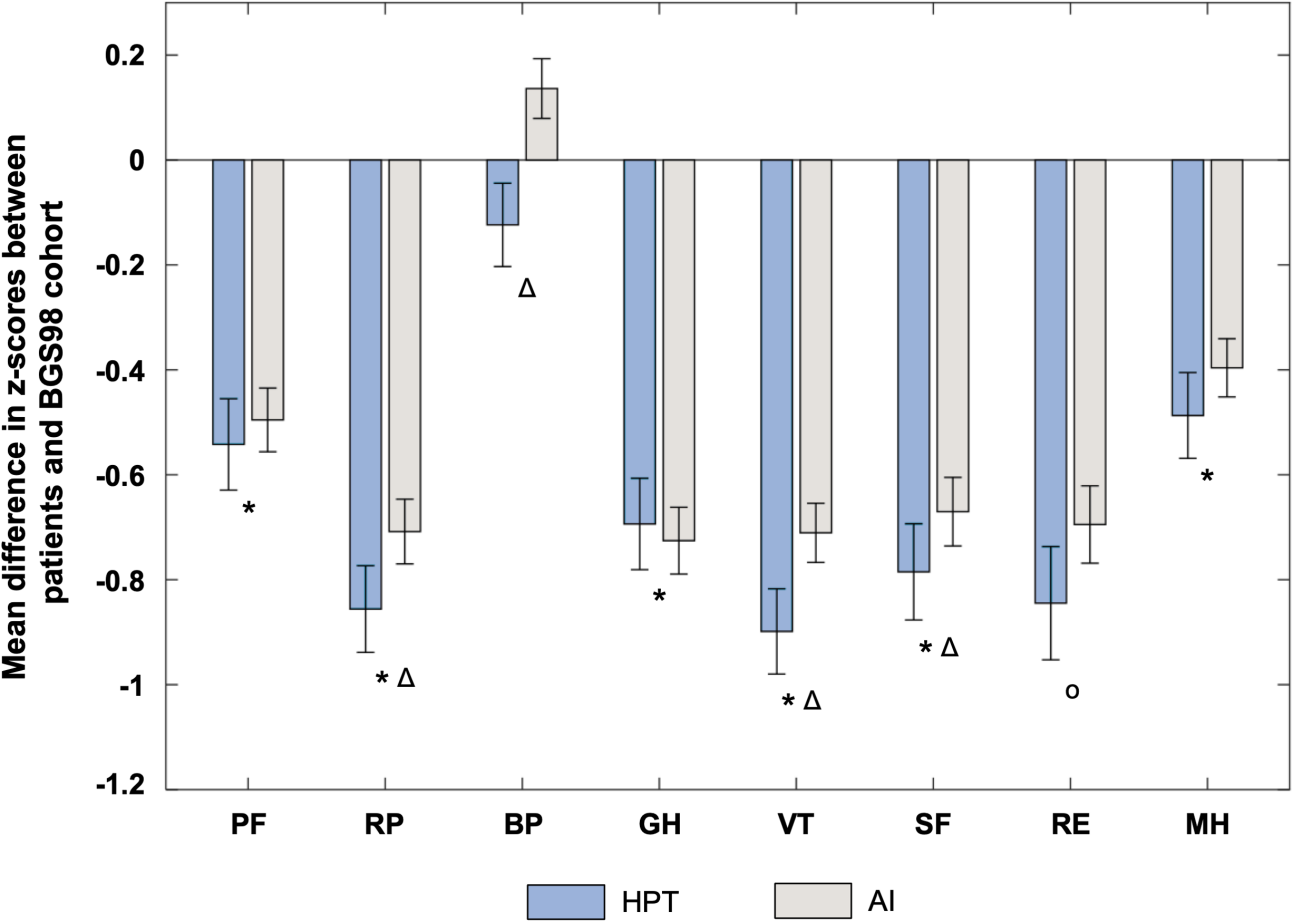
Subjective health status of patients with hypoparathyroidism (HPT, n=205) and patients with adrenal insufficiency (AI, n=214). Illustrated are differences in z-scores (mean ± SEM) for the SF-36 domains in comparison with normative data from the general German population (BGS98, n=7124). *p<0.001 HPT compared to normative data from BGS98, o p<0.05 HPT compared to normative data from BGS98, Δ p<0.05 HPT compared to AI. PF, physical functioning; RP, role limitations due to physical problems; BP, bodily pain; GH, general health perception; VT, vitality; SF, social functioning; RE, role limitations due to emotional problems; MH, general mental health.

In 7 out of 8 domains of the SF-36, patients with HypoPT had worse values than controls from the general population. Surprisingly, impairment of quality of life was also significantly more severe in 4 out of 8 domains compared to patients with adrenal insufficiency a potentially life-threatening disease in which reduced subjective health status is well documented (27, 28). Those domains included role limitations because of physical problems (p=0.002), bodily pain (p<0.001), vitality (p=0.001) and social functioning (p=0.018) (**Figure 3**).

### Impact on daily life

**Figure 4:**
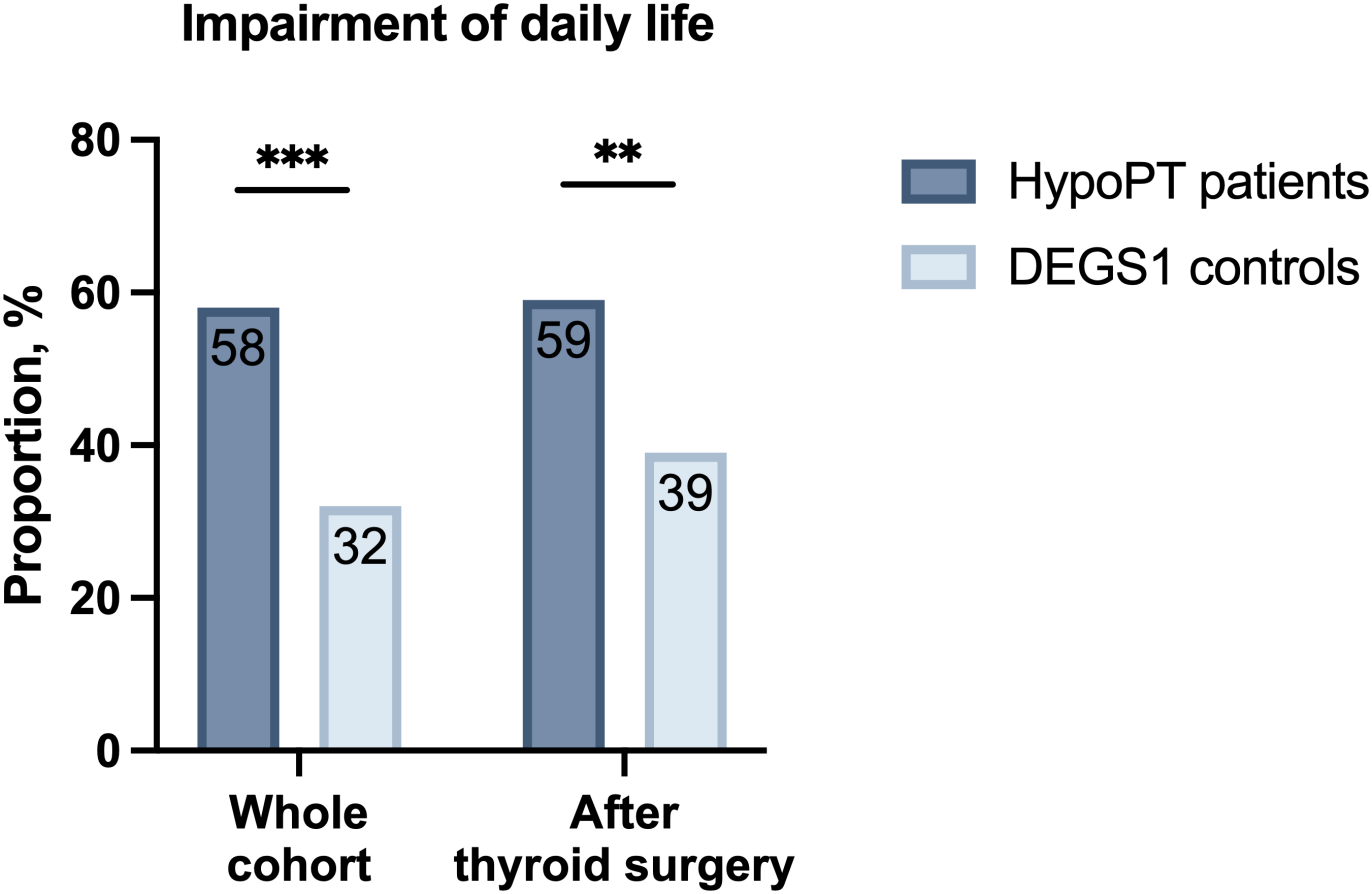
Perception of general health status of patients with hypoparathyroidism (HypoPT) compared to controls from DEGS1. Whole sample: n=202 HypoPT patients and 4040 DEGS1 controls; After thyroid surgery: n= 162 HypoPT patients and 162 DEGS1 controls. **p<0.005, ***p<0.001.

**Figure 5:**
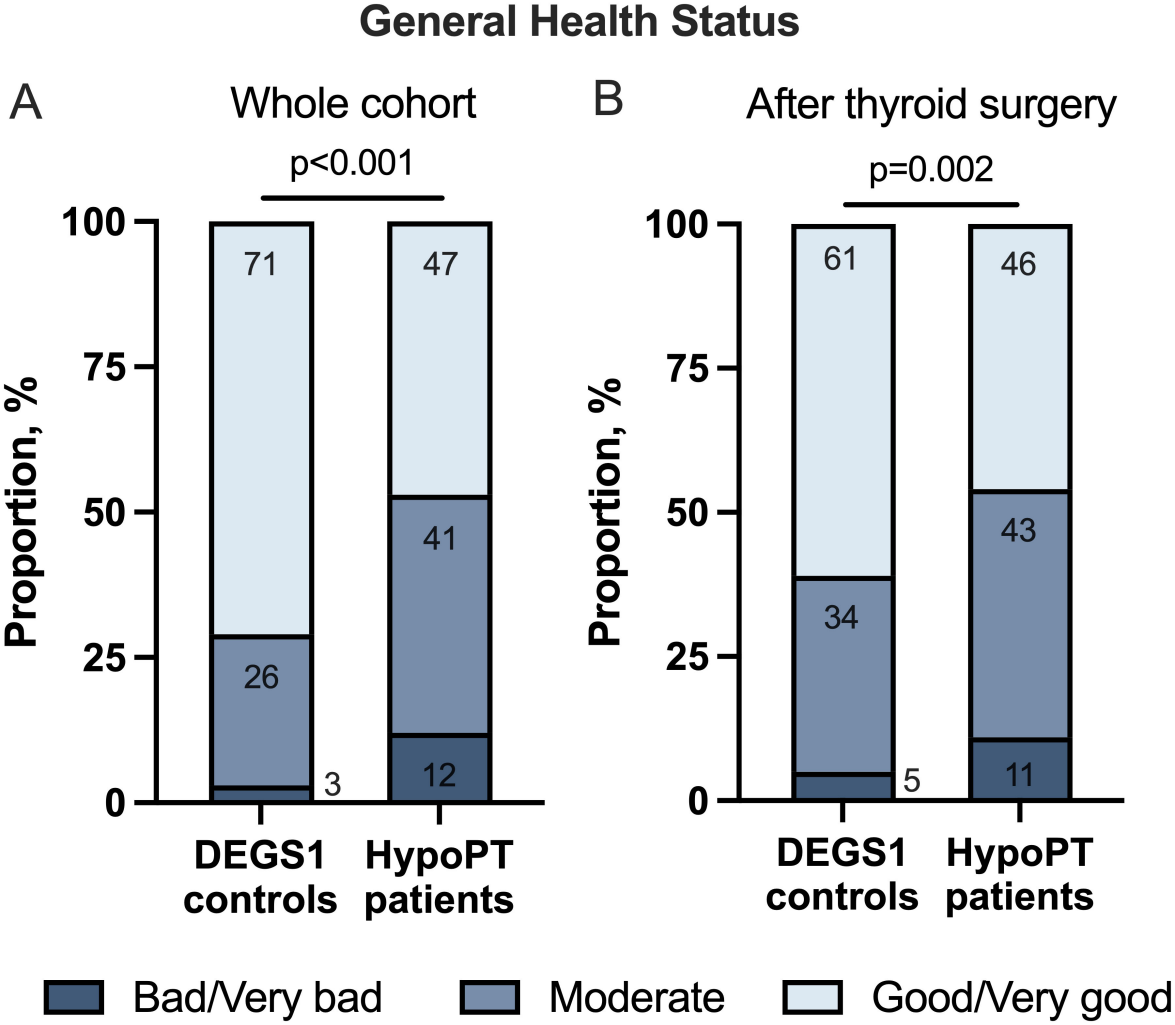
**(A)** Impairment of daily life of patients with hypoparathyroidism (HypoPT, n=202) compared to controls from DEGS1 (n=4040), as well as **(B)** impairment of daily life of patients with hypoparathyroidism due to thyroid surgery (n=162) compared to controls from DEGS1 after thyroid surgery (n=162).

Rating of general health status was significantly worse in patients with HypoPT compared to controls from DEGS1 (p<0.001) (**Figure 4**). Additionally, a significantly stronger impairment of daily life activities for health reasons was found (58% vs. 32%, p<0.001) (**Figure 5**). Comparing patients with postoperative HypoPT to controls after thyroid surgery also showed significantly worse rating of general health status in the HypoPT group independently of the reason for thyroid surgery (11% vs. 5%, p=0.015) (**Figure 4**). Furthermore, patients with HypoPT after thyroid surgery reported more often an impairment of daily life activities than controls (59% vs. 39%, p=0.001) (**Figure 5**).

37% of HypoPT patients younger than 65 years reported impairment of work life and for 32% this resulted in consequences regarding their work activity (14% out of work, 10% reduced working hours, 3% job change, 7% other). In comparison to the control cohort, significantly more patients affected by HypoPT retired at an age below 65 years (21% vs. 10%, p<0.001). However, percentage of part time work and days missing at work did not differ among cohorts (data not shown).

## Discussion

One major result of this study is the significantly reduced SHS in patients with HypoPT compared to sex- and age-matched controls from the general German population, but also to patients with adrenal insufficiency. This result was obtained despite established replacement therapy for HypoPT and was independent of the etiology of HypoPT, treatment facility and membership in a self-help network. These observations not only corroborate findings of previous studies bearing evidence of reduced SHS among HypoPT patients (5, 15, 16), but also provide further insight on the severity of their impairment. However, patients with non-surgical and pseudo-HypoPT scored worse in only five or four domains, respectively. This observation may be explained by the small number of patients. Worse scoring in four domains of SF-36 of HypoPT patients compared to patients with AI suggests an even greater interference of HypoPT regarding SHS (27). Calculating age- and sex-adjusted z-scores for both patient groups allowed us to exclude a bias caused by the varying age- and sex distribution among the cohorts. The clinical relevance of reduced SHS is reflected by an impairment of daily life and work life in terms of occupational changes and a higher rate of retired subjects at an age below 65 years compared to controls. These findings are in accordance with a study among U.S. residents with HypoPT showing a significant interference with life in 45% and a disease-associated change in employment status in 20% of patients (6). Büttner et al. furthermore reported significant impact on work ability in 52% of patients with HypoPT (29). The high number of experienced clinical symptoms within the last 12 months may have had an influence on impairment of SHS as participants with more symptoms showed stronger impairment. Our data also confirms that not thyroid surgery itself but HypoPT accounts for the impairment in daily life activities (15, 27).

As conventional treatment with vitamin D and calcium does not fully restore SHS, it is conceivable that this is directly caused by the lack of PTH. Previously, investigation of the effect of therapeutic regimens with PTH on SHS has shown inconsistent results (30–32). More recently, recombinant PTH therapy has been shown to improve quality of life in adults with chronic hypoPT, particularly in patients with impaired baseline scores. Randomized controlled trials, long-term extension studies, and meta-analyses report clinically meaningful improvements in both physical and mental health domains. In several cohorts, these benefits were observed within weeks to months after treatment initiation and were sustained over extended follow-up periods (33–35). In this survey the very small number of patients treated with PTH did not allow valid comparisons.

Moreover, SHS may be influenced by higher lifetime prevalence of psychiatric comorbidities compared to the general population. The significantly higher occurrence of anxiety among patients with postsurgical HypoPT compared to controls after thyroid surgery suggests that HypoPT itself contributes to changes in fear perception. However, contrary to the findings of this survey, Danish HypoPT patients also showed an increased risk of depression compared to the general population, whereas the risk of being hospitalized because of anxiety disorders was not significantly higher (10, 11). As the PTH2 receptor was found in several regions of the central nervous system, impaired SHS and a higher incidence of psychiatric comorbidities may result from a direct lack of PTH in the amygdala (36–38).

Another major result of this survey is the increased risk for HypoPT associated comorbidities as well as the high rate of symptomatic episodes and hypocalcemic emergencies. Standard treatment of HypoPT does not fully restore calcium/phosphorus homeostasis and increases the risk of hypercalciuria, which may result in a higher risk of renal insufficiency, renal stones and nephrocalcinosis. Lifetime prevalence of renal insufficiency was significantly higher in HypoPT compared to controls from the general German population, corroborating earlier studies (8, 14, 39). Additionally, 8% and 6% of participants reported kidney calcification in terms of renal stones and nephrocalcinosis, respectively. Moreover, a high number of participants (18%) were affected by cataract. Consistent with our findings in previous studies a high prevalence of cataract in HypoPT was reported. While previous studies suggest a higher prevalence of comorbidities with increased disease duration (10, 11), we were not able to observe such correlation.

The intake of antihypertensive medication and medication affecting the cardiovascular system was significantly increased in comparison to the general population. This increase is not only due to increased prescription of diuretics, as some patients may be treated with thiazide diuretics to decrease urinary calcium excretion. Furthermore, significantly more patients were treated with beta blockers and medication targeting the renin-angiotensin-aldosterone system, suggesting a higher prevalence of hypertension. The increased use of antihypertensive medication likely reflects a higher prevalence of hypertension in HypoPT, as recently confirmed by objective data (40). This is likely multifactorial: conventional replacement with calcium and active vitamin D increases the risk of hypercalciuria and subsequent renal impairment, both established drivers of secondary hypertension. Additionally, the loss of PTH-mediated vasodilation via the PTH-1 receptor may increase peripheral vascular resistance. These findings support the view that conventional therapy primarily targets calcium homeostasis but falls short of restoring the full spectrum of PTH’s systemic physiological actions.

The increased use of antiepileptic drugs, potentially suggesting a higher rate of seizures in patients affected by HypoPT, may be caused by an increased neuromuscular excitability due to hypocalcemia. However, the exact pathomechanism is still unknown.

To our knowledge, this study is the first to investigate potential trigger factors of hypocalcemic events. As physical activity, hot weather, infectious diseases and perspiration were frequently observed trigger factors, situations with high electrolyte loss may provoke hypocalcemic symptoms. Few studies investigated emergency events and hospitalization rate in HypoPT patients. Since primary diagnosis 36% of our patients had presented at least once at an emergency department or were admitted to hospital because of severe symptoms caused by HypoPT. 36% of patients required at least once i.v. calcium due to severe hypocalcemia. In a web-based survey in the U.S. a higher rate (79%) of patients reported hospital stays or emergency department visits due to HypoPT; however, 64% were members of a self-help organization and therefore may be affected more severely (6). Furthermore, our percentage of patients requiring emergency treatment of hypocalcemia is higher than previously reported (8), most likely because previous reports excluded the first 30 days after diagnosis of HypoPT from the analysis. The present survey did not evaluate the specific reasons for visits in emergency departments or hospital stays and the trigger factors for hypocalcemic emergency events. These aspects should be investigated in further studies. However, the general hospitalization rate of HypoPT patients in the past 12 months was not significantly different from controls of the general German population.

### Strengths and limitations

The major strengths of the present study are the multicenter design with a large sample size and the availability of well-matched representative controls. In addition, the survey population included only a limited proportion of members of Self-Helping groups, reducing the likelihood of a selection bias. However, the study also has some limitations. First, the response rate to our survey was only 52%, and might bear a selection bias. However, the total number of more than 200 patients, who provided a very detailed feedback with data on multiple questionnaires is still remarkable. More importantly, the various morbidities assessed in our study are based on self-report and not on clinical examination. Accordingly, these numbers should be regarded as minimum estimates and may have been higher if systematical assessments were performed. The use of generic quality-of-life instruments instead of a disease-specific questionnaire may have limited sensitivity to detect certain HypoPT-specific symptoms. Furthermore, there was no correlation between disease duration and the different comorbidities. This may be caused by an insufficient number of individual comorbidities. Also laboratory measurements (e.g. PTH, calcium, phosphorus) were unavailable at the time of study design, leaving the possibility of undetected associations. Lack of biochemical parameters did not allow us to distinguish between well-controlled and insufficiently controlled HypoPT patients. However, the high proportion of reported hypocalcemic symptoms, emergency department visits, and intravenous calcium administrations suggests that a substantial subset of patients experienced clinically relevant instability despite standard therapy. Importantly, most patients in this cohort were followed in specialized endocrine centers, indicating that even under routine endocrine care and conventional treatment strategies, optimal disease control may not be consistently achieved. While biochemical data would have allowed more precise interpretation, the frequency of symptomatic events itself represents a meaningful patient-centered outcome and underscores the persistent burden of disease under current standard-of-care management. The comparison cohorts were derived from population-based studies conducted during different time periods. Although age- and sex-matching and standardized instruments were used, a potential historical bias cannot be completely excluded. Furthermore, HypoPT was not specifically assessed in the DEGS1 cohort. Although the overall prevalence in the general population is low, a residual inclusion of patients with undiagnosed HypoPT among controls after thyroid surgery cannot be entirely excluded. We acknowledge that pseudo-HypoPT is not classified as hypoparathyroidism in current clinical guidelines. Therefore, inclusion of pseudo-HypoPT patients represents a limitation of this study. However, given the small number of pseudo-HypoPT patients and the absence of significant differences in subjective health status across etiological subgroups, a relevant influence on the overall results appears unlikely. Finally, the number of patients with non-surgical HypoPT was low, which prevented us from drawing robust conclusions on differences between the various entities of HypoPT.

In conclusion, chronic HypoPT is associated with a significantly reduced SHS compared to the general population, but also to patients with AI despite standard replacement therapy, resulting in a strong impairment of daily life and work life. Furthermore, patients suffer from a variety of symptoms, comorbidities and a high number of HypoPT associated emergencies.

## Data Availability

The raw data supporting the conclusions of this article will be made available by the authors, without undue reservation.
