# Supplementary material for: Subjective health status, life performance and complications in chronic hypoparathyroidism – a German multicenter survey
Source: Front Endocrinol (Lausanne). 2026 Mar 31;17:1723640. doi: 10.3389/fendo.2026.1723640 (PMC13076148; doi:10.3389/fendo.2026.1723640)
Supplement: Supplementary file 1 [file DataSheet1.pdf]

<u>Patientenfragebogen</u> zur Krankheitslast und zum Auftreten von hypocalcämischen Krisen bei Patienten mit chronischem Hypoparathyreoidismus

<u>Datum der Befragung:</u> _ _/ _ _/ 20_ _

1. Bitte geben Sie Ihre Personalien an:	Vorname ____________<br>Nachname ____________<br>Geburtsdatum ________ *(tt.mm.jjjj)*<br>Geschlecht ○ männlich ○ weiblich<br>Straße ____________<br>PLZ, Ort ____________<br>Telefonnr. ____________<br>E-Mail-Adresse ____________
2. Bei welchem Institut sind Sie aufgrund Ihres Hypoparathyreoidismus in Behandlung?	○ Universitätsklinikum Würzburg<br>○ Sonstiges: ____________
3. Bei welchem Arzt befinden Sie sich aufgrund Ihres Hypoparathyreoidismus in Behandlung?	Vorname ____________<br>Nachname ____________<br>Straße ____________<br>PLZ, Ort ____________<br>Telefonnr. ____________
4. Wann wurde die Diagnose eines Hypoparathyreoidismus gestellt?	________ *(Monat/ Jahr)*
5. Sind Sie Mitglied in einer Selbsthilfegruppe aufgrund Ihres Hypoparathyreoidismus ?	○ ja ○ nein
6. An welcher Form des Hypoparathyreoidismus leiden Sie?	○ erworben (nach Operation, bestrahlungsinduziert, autoimmunes polyglanduläres Syndrom, Hämochromatose)<br>○ angeboren (isolierte/ syndromale Nebenschilddrüsen-Aplasie)<br>○ Pseudohypoparathyreoidismus<br>○ unbekannt

7. Auf welche der folgenden Ursachen ist Ihr Hypoparathyreoidismus zurückzuführen?	○ Nach Operation, aufgrund<br>○ Struma (Kropf)<br>○ mit Knoten<br>○ ohne Knoten<br>○ Schilddrüsen-Krebs<br>○ Morbus Basedow<br>○ Nebenschilddrüsen-Adenom<br>○ Nebenschilddrüsen-Hyperplasie<br>○ Nebenschilddrüsen-Krebs<br>○ Neck-dissection<br>○ Sonstiges: ________________________________<br>○ Isolierte Nebenschilddrüsen-Aplasie<br>○ Syndromale genetische Erkrankung<br>○ autoimmunes polyglanduläres Syndrom<br>○ Hämochromatose<br>○ Bestrahlung<br>○ Parathormon-Resistenz (Pseudohypoparathyreoidismus)<br>○ unbekannt<br>○ Sonstiges: ________________________________
8. Sind Sie berufstätig?	○ ja ○ nein ○ berentet<br>○ Sonstiges: ________________________________<br>**Wenn ja**,<br>1) An wie vielen Arbeitstagen **in den letzten 12 Monaten** waren Sie durch einen Arzt arbeitsunfähig geschrieben?<br>*(Bitte auch die Tage berücksichtigen, die Sie im Krankenhaus gelegen haben)*<br>○ ungefähr ______ Arbeitstage<br>○ an keinem Tag<br>2) Welche Art der Erwerbstätigkeit trifft zu?<br>○ Vollzeit (>35 Stunden)<br>○ Teilzeit (15-34 Stunden)<br>○ Teilzeit (<15 Stunden)
9. Sind Sie durch Ihren Hypoparathyreoidismus beruflich eingeschränkt?	○ ja ○ nein<br>○ nicht bekannt, da Krankheit bereits vor Berufsbeginn bestand<br>**Wenn ja**, welche beruflichen Konsequenzen haben sich dadurch ergeben?<br>○ keine<br>○ Ich arbeite nicht mehr.<br>○ Ich arbeite mit reduzierter Stundenzahl.<br>○ Ich musste meinen Beruf wechseln.<br>Neuer Beruf: ________________________________<br>○ Sonstiges: ________________________________

10. Kam es **in den letzten 12 Monaten** zu einem oder mehreren der nachfolgend aufgeführten Beschwerden?	○ ja ○ nein<br>**Wenn ja**, bitte näher erläutern:<br>1) Beschwerden *(Mehrfachnennungen möglich)*<br>☐ Kribbeln, Taubheitsgefühl, Kälte-/ Wärmewahrnehmungsstörung in Körperregionen (z.B.: der Hände, im Bereich des Mundes)<br>☐ Muskelkrämpfe der Hände und Füße<br>☐ Krampfanfall<br>☐ Muskelschmerzen<br>☐ Muskelschwäche<br>☐ Knochenschmerzen<br>☐ Atemnot<br>☐ Darmkrämpfe<br>☐ unerklärliche Angstgefühle<br>☐ gesteigerte Erregbarkeit<br>☐ Verwirrtheit<br>☐ Sonstiges: ____________________<br>2) Welche Behandlung wurde daraufhin veranlasst? *(Bitte nur eine Angabe)*<br>○ keine ärztliche Behandlung ○ ambulante ärztliche Behandlung<br>○ stationäre Behandlung im Krankenhaus ○ Aufnahme auf Intensivstation
11. Mussten Sie schon einmal aufgrund Ihres Hypoparathyreoidismus in die **Notaufnahme** oder in das **Krankenhaus** aufgenommen werden?	○ ja ○ nein<br>**Wenn ja**,<br>Wann war dies **das letzte Mal** nötig gewesen?<br>○ In den **letzten 12 Monaten**<br>○ In den **letzten 24 Monaten** (aber nicht in den letzten 12 Monaten)<br>○ Vor mehr als 24 Monaten
12. Wie oft mussten Sie **seit der Erstdiagnose** des Hypoparathyreoidismus aufgrund einer Verschlechterung intravenös (Infusion) mit Calcium behandelt werden?	○ noch nie ○ einmal ○ zweimal<br>○ dreimal ○ viermal ○ fünfmal<br>○ sechsmal ○ häufiger, bitte Anzahl angeben: ______
13. Wie viele Stunden pro Tag verspüren Sie Symptome Ihres Hypoparathyreoidismus ?	________ Stunden pro Tag

14. Wie viele Arztbesuche waren aufgrund Ihres Hypoparathyreoidismus **in den letzten 12 Monaten** nötig?	________ Anzahl der Arztbesuche in den letzten 12 Monaten
15. Wie viele Blut-Kontrollen des Calciumwertes waren **in den letzten 12 Monaten** nötig?	________ Calciumwert-Kontrollen in den letzten 12 Monaten
16. Gibt es Situationen, in denen sich die **Symptome** Ihres Hypoparathyreoidismus **verstärken**?	○ ja ○ nein<br>**Wenn ja**,<br>1) in welchen Situationen geschieht dies? *(Mehrfachnennungen möglich)*<br>☐ Sportliche Aktivität<br>☐ Vermehrtes Schwitzen<br>☐ Hitze<br>☐ Infekte<br>☐ Magen-Darm-Beschwerden<br>☐ Sonstige: ____________________

	sehr gut	gut	mittelmäßig	schlecht	sehr schlecht
1. Wie zufrieden sind Sie mit Ihrer derzeitigen Behandlung des Hypoparathyreoidismus?	○	○	○	○	○

2. An welchen der folgend genannten Erkrankungen leiden Sie? *(gegebenenfalls bitte auch das ungefähre Datum der Erstdiagnose angeben)*	Erstdiagnose *(Monat/ Jahr)*
☐ Nierensteine	________
☐ Niereninsuffizienz	________
☐ Verkalkung der Nieren (Nephrocalcinose)	________
☐ Herzrhythmusstörungen	________
☐ Krampfanfälle	________
☐ Grauer Star (Katarakt)	________
☐ Depression	________
☐ Angststörung	________

3. Leiden Sie an sonstigen chronischen Erkrankungen? *(gegebenenfalls bitte auch das ungefähre Datum der Erstdiagnose angeben)*	○ ja ○ nein<br>**Wenn ja**, bitte erläutern:<br>Erkrankung ____ Erstdiagnose *(Monat/ Jahr)* ____

4. Welche Medikamente nehmen Sie ein? (Bitte **alle** auflisten, einschließlich Medikamenten für andere Erkrankungen, Calcium, Magnesium und sonstige Nahrungsergänzungsmitteln)

Medikamentenname	Dosis pro Portion (z.B. je 1 Tablette)	Art der Einnahme (z.B. als Tablette, Spritze, etc.)	Einnahmeschema (z.B. 1-0-2-0, wenn Sie eine Tablette morgens und 2 Tabletten abends einnehmen) morgens	mittags	abends	nachts	Bei Bedarf
1)							☐
2)							☐
3)							☐
4)							☐
5)							☐
6)							☐
7)							☐
8)							☐
9)							☐
10)							☐
11)							☐
12)							☐

5. Sind Sie **während der letzten 12 Monate** zur stationären Behandlung im Krankenhaus gewesen?	○ ja ○ nein<br>**Wenn ja,** bitte näher erläutern<br>1) Wie oft waren Sie **in letzten 12 Monaten** im Krankenhaus?<br>_________ Anzahl Krankenhausaufenthalte<br>2) Wie viele Tage haben Sie insgesamt **in den letzten 12 Monaten** im Krankenhaus zugebracht?<br>_________ Tage
6. Wie gut kommen Sie mit dem Hypoparathyreoidismus zurecht?	sehr gut ○ / gut ○ / mittelmäßig ○ / schlecht ○ / sehr schlecht ○
7. Fühlen Sie sich mit der Medikamenten-einnahme überfordert?	überhaupt nicht ○ / etwas ○ / mäßig ○ / ziemlich ○ / sehr ○
8. In welchem Ausmaß sind Sie durch Ihre Krankheit in der Ausübung Ihrer alltäglichen Tätigkeiten eingeschränkt?	○ erheblich eingeschränkt<br>○ eingeschränkt<br>○ nicht eingeschränkt<br>○ weiß ich nicht
9. Wie ist Ihr Gesundheitszustand im Allgemeinen?	sehr gut ○ / gut ○ / mittelmäßig ○ / schlecht ○ / sehr schlecht ○

10. Hatten Sie **in den letzten 12 Monaten** schon einmal einen Angstanfall- manche nennen es auch Panikattacke oder Angstattacke-, bei dem Sie ohne Grund plötzlich von einem Gefühl starker Angst, Beklommenheit oder Unruhe überfallen wurden?

○ ja ○ nein

11. Gab es **in den letzten 12 Monaten** schon einmal eine Zeitspanne **von einem Monat oder länger**, in der Sie sich häufig ängstlich, angespannt und voller Besorgnis gefühlt haben?

○ ja ○ nein

12. Haben Sie **in den letzten 12 Monaten** über **mehr als 2 Wochen** fast täglich unter Gefühlen von Traurigkeit oder Niedergeschlagenheit gelitten?

○ ja ○ nein

13. Litten Sie **in den letzten 12 Monaten** über **mehr als 2 Wochen** fast täglich unter Interessenverlust, Müdigkeit oder Energieverlust?

○ ja ○ nein

14. Wie oft hatten Sie in den **vergangenen 4 Wochen** aufgrund Ihrer **körperlichen Gesundheit** irgendwelche Schwierigkeiten bei der Arbeit oder anderen alltäglichen Tätigkeiten im Beruf bzw. zu Hause?

	immer	meistens	manchmal	selten	nie
1) Ich konnte nicht so lange wie üblich tätig sein.	○	○	○	○	○
2) Ich habe weniger geschafft, als ich wollte.	○	○	○	○	○
3) Ich konnte nur bestimmte Dinge tun.	○	○	○	○	○
4) Ich hatte Schwierigkeiten bei der Ausführung (z.B. ich musste mich besonders anstrengen).	○	○	○	○	○

15. Wie oft hatten Sie in den **vergangenen 4 Wochen** aufgrund **seelischer Probleme** irgendwelche Schwierigkeiten bei der Arbeit oder anderen alltäglichen Tätigkeiten im Beruf bzw. zu Hause (z.B. weil Sie sich niedergeschlagen oder ängstlich fühlten)?

	immer	meistens	manchmal	selten	nie
1) Ich konnte nicht so lange wie üblich tätig sein.	○	○	○	○	○
2) Ich habe weniger geschafft als ich wollte.	○	○	○	○	○
3) Ich konnte nicht so sorgfältig wie üblich arbeiten.	○	○	○	○	○

16. Wie sehr haben Ihre körperliche Gesundheit oder seelischen Probleme in den **vergangenen 4 Wochen** Ihre normalen Kontakte zu Familienangehörigen, Freunden, Nachbarn oder zum Bekanntenkreis beeinträchtigt?

überhaupt nicht	etwas	mäßig	ziemlich	sehr
○	○	○	○	○

Vielen Dank für Ihre Mitarbeit!
